# Real-world analysis of immune checkpoint inhibitor efficacy and response predictors in patients treated at the CCCMunich^LMU^ outpatient clinic

**DOI:** 10.1038/s41598-025-30220-0

**Published:** 2025-12-08

**Authors:** Klara Dorman, Kilian Breitenwieser, Laura Fischer, Danmei Zhang, Victoria Probst, Lena Weiss, Kathrin Heinrich, Wolfgang G. Kunz, Julian W. Holch, Clemens Gießen-Jung, Michael Haas, Stefan Boeck, Michael von Bergwelt-Baildon, Timotheus Landfarth, Roman Hornung, Jozefina Casuscelli, Karin Berger-Thürmel, Volker Heinemann, C. Benedikt Westphalen, Anna Reischer

**Affiliations:** 1 Department of Medicine III and Comprehensive Cancer Center, University Hospital, Ludwig-Maximilians-Universität München, Marchioninistr. 15, 81377 Munich, Germany; 2 German Cancer Consortium (DKTK), Partner Site Munich, Munich, Germany; 3 Department of Radiology, University Hospital, LMU Munich, Munich, Germany; 4 Department of Hematology and Oncology, München Klinik Neuperlach, Munich, Germany; 5 Max Planck Institute of Psychiatry, Munich, Germany; 6 Institute for Medical Information Processing, Biometry and Epidemiology, Faculty of Medicine, LMU Munich, Munich, Germany; 7 Munich Center for Machine Learning (MCML), Munich, Germany; 8 Department of Urology, University Hospital, LMU Munich, Munich, Germany

**Keywords:** Immune checkpoint inhibitors, Solid tumors, Adverse events, Immunotherapy, Autoimmune disorders, Response predictors, Cancer, Diseases, Immunology, Oncology

## Abstract

Prediction of response to checkpoint inhibition (ICI) and immune related adverse events (irAEs) remain a challenge in the immunotherapy of solid tumors. To better understand the real-world clinical courses under ICI and identify possible predictive markers for response and irAEs, we retrospectively collected and analysed data from our interdisciplinary CCCMunich^***LMU***^ outpatient clinic. We analysed the clinical course, standard laboratory parameters and staging results of 342 patients who received ICI therapy in the interdisciplinary outpatient clinic of the CCCMunich^***LMU***^ between January 2015 and November 2020. Initial response to ICI was defined as complete response, partial response, stable disease or mixed response with treatment continuation in the second radiologic staging after start of therapy. Median age of the included patients was 67 years (25–89). More male patients received ICI on our outpatient ward than female patients (67.8% vs. 32.2%). The most common entities were urothelial carcinoma (21.3%) and bronchial carcinoma (19.6%), followed by renal cell carcinoma (18.7%), and head and neck tumors (10.8%). 24.6% of patients were initial responders. Patients who did not respond to ICI therapy had lower leukocyte, lymphocyte, monocyte, and neutrophil counts before treatment start compared to initial responders. The consolidation of these laboratory parameters into a score could not accurately predict initial response or progression-free survival (PFS). IrAEs occurred in approximately one-third of all included patients. The main side effects in patients expierencing irAEs were thyroiditis (20.5%), pneumonitis (16.4%), hepatitis (15.8%), and dermatological side effects (11.0%). Patients experiencing irAEs had a significantly longer PFS than patients without irAEs. Side effects were more frequent and severe in patients treated with combined ICI. Autoimmune disorders were not associated with more frequent occurrence of irAEs. This retrospective analysis illustrates the real-world use of ICI in clinical practice. With only 24.6% of patients responding to ICI, the clinical need for predictive markers is obvious and standard laboratory parameters such as white blood cell counts may become part of a comprehensive score to predict response and adverse events.

**Supplementary Information:**

The online version contains supplementary material available at 10.1038/s41598-025-30220-0.

## Introduction

Immune checkpoint inhibitors (ICI) have revolutionized the treatment landscape of many solid tumors and have become an important pillar in various standard treatment protocols. However, not all patients benefit from ICI. Response rates range from 40 to 70% in certain cancers like malignant melanoma and Hodgkin’s disease, whereas the response rates for most other advanced cancers are only 10–25%^1^. Therefore, a large group of patients is subjected to the risk of immune related adverse events (irAE) without betterment of their malignant disease. While an irAE may present as only a mild rash or manageable thyroid disorders, the wide range of possible irAEs also includes pneumonitis, hepatitis, and myocardits with potentially fatal outcome^2,3^. As soon as higher-grade irAEs occur, immunosuppressive therapy with corticosteroids or other immunosuppressive drugs has to be initiated. This again carries the risk of additional side effects. Furthermore, the occurrence of an irAE can lead to a delay of the tumor-directed treatment due to the necessary interruption or discontinuation of the ICI.

Prediction of irAEs and response to ICI remain a challenge. In some solid tumor entities, FDA and EMA approvals are restricted to tumors with presence of certain predictive markers. Common markers currently used for response prediction include programmed death-ligand 1 (PD-L1) expression, tumor mutational burden (TMB), and microsatellite instability^4–10^. However, these biomarkers are not sufficient to accurately predict response in all patients. Some patients respond to ICI therapy without corresponding biomarker evidence, and vice versa.

Among others, the composition of immune cells and measurement of cytokines in the peripheral blood have been investigated as possible markers for prediction of irAEs. However, sensitive and specific markers are still lacking^11^. A possible risk factor for the occurrence of irAEs may be preexisting autoimmune disorders. A worsening of the preexisting disease or other irAEs have been described in a large proportion of these patients when treated with ICIs^12,13^. Since patient selection criteria in clinical ICI trials commonly exclude patients with autoimmune disorders, definitive conclusions are limited and commonly based on real world data.

While clinical trials yield important data on the efficacy and safety of ICIs under controlled conditions in selected patients, real-world experience provides complementary insights into their effectiveness and tolerability in broader, more heterogeneous populations. We retrospectively collected and analysed data from our interdisciplinary CCCMunich^LMU^ outpatient clinic to comprehensively evaluate the real-world clinical courses under ICI and identify possible predictive markers for response and irAEs.

## Patients and methods

### Patient population and data collection

In this retrospective single-center cohort study, patients with hematologic and oncologic malignancies receiving ICI at the interdisciplinary outpatient clinic at CCCMunich^LMU^ between 1st of January 2015 and 1st of November 2020 were included. Patients who did not continue treatment at our center shortly after ICI initiation were excluded due to lack of information on the further clinical course. Data essential to the study, including demographic information, clinical history, laboratory and imaging results, treatment modalities and outcomes were extracted from the patients ‘ medical charts. Laboratory results included leukocyte count, lymphocyte count, monocyte count, neutrophil count, LDH, neutrophil–lymphocyte ratio (NL-ratio), C-reactive protein (CRP), eosinophil count, serum magnesium, serum protein, and serum albumin. Baseline characteristics were collected from reports closest to first ICI treatment. The patients were divided into two groups based on their response according to RECIST v1.1 in the second radiological staging. Patients with a complete remission (CR), a partial response (PR), stable disease (SD), or mixed response (MR) with treatment continuation were assigned to the “Initial Responder” (IR) group. The remaining patients were assigned to the "Initial Non-Responder" (INR) group. Progression-free survival (PFS) was defined as the time from ICI initiation to documented disease progression, as determined by RECIST version 1.1 criteria, or death from any cause, whichever occurred first. Patients without progression or death at the time of analysis were censored at the date of their last follow-up. Imaging took place within clinical routine approximately every three months. IrAEs were classified according to the Common Terminology Criteria for Adverse Events (CTCAE) Version 5.0. The study had been approved by the local ethics committee of the Ludwig-Maximilians-University Munich (20-1161). Due to the retrospective nature of the study, the local ethics committee of the Ludwig-Maximilians-University Munich waived the need of obtaining informed consent.

### Statistical analysis

Statistical analysis was performed using SPSS Version 29, Graph Pad Prism Version 9, and R Version 4.2.3. Survival probabilities for time-to-event endpoints were estimated using the Kaplan–Meier method. Group differences in survival were evaluated using the log-rank test. Differences in categorical variables between groups were assessed using the Chi-squared test. Differences in continuous variables, including comparisons between patients with and without irAEs and analyses of laboratory parameters, were analyzed using the Mann–Whitney U test. The significance level was set at α = 0.05.

All univariate subgroup and laboratory comparisons were considered exploratory, and nominal p-values are reported without adjustment for multiple testing.

To develop predictive models for the risk of disease progression based on ten potential predictors, Cox proportional hazards regression with PFS as the outcome was employed using forward selection. The ten potential predictors were leukocyte count, lymphocyte count, neutrophil count, eosinophil count, monocyte count, LDH, CRP, magnesium, protein, and albumin. Because all candidate variables had missing values (ranging from 16.3% to 66.4% for leukocytes and eosinophilic granulocytes respectively), multiple imputation by predictive mean matching was performed^14^. The data were imputed M = 20 times. Multiple imputation assumes that data are missing at random (MAR), which appears plausible in this context, since the availability of laboratory measurements was not dependent on patient characteristics or outcomes, but rather the attending physician or nursing staff.

The resulting 20 imputed datasets were then combined (“stacked”), and forward selection was carried out on the merged dataset. Two models were generated, one based on the Akaike Information Criterion (AIC) and one based on the Bayesian Information Criterion (BIC). The AIC generally selects more variables than the BIC, as it applies a less stringent penalty for including additional predictors. To account for the fact that each individual appeared M times in the stacked dataset and for the fact that part of the data was missing, the log-likelihood was adjusted by multiplying it by the factor (1 − f)/M, where f denotes the overall proportion of missing values in the dataset^15,16^.

We used this stacked multiple imputation approach because variable selection was required among the ten candidate predictors. Applying Rubin’s rules, which are commonly used in multiple imputation settings, would necessitate identical model structures across all imputed datasets, which is generally not compatible with variable selection performed independently within each dataset. The stacked approach, as described above, enables a unified selection procedure across the imputed datasets while appropriately accounting for repeated observations and missingness. Moreover, only 28 of the 342 patients had complete data across all ten candidate predictors. As a complete-case analysis would therefore rely on less than 10% of the cohort and could yield biased and unstable estimates, it was not performed.

For model validation, fivefold cross-validation repeated ten times was performed. Multiple imputation was conducted separately for the training and test data within each cross-validation iteration^17^, again using M = 20 imputations. Model building was carried out on the stacked imputed training data using forward selection, applying the same correction for repeated observations and missingness as described above.

Model performance was evaluated in each cross-validation iteration across all 20 imputed test datasets, and the resulting performance metrics were averaged over the 20 datasets. The concordance index (for discrimination) and the Brier score (for calibration and discrimination) were used as performance measures.

## Results

### Patient population

**Fig. 1:**
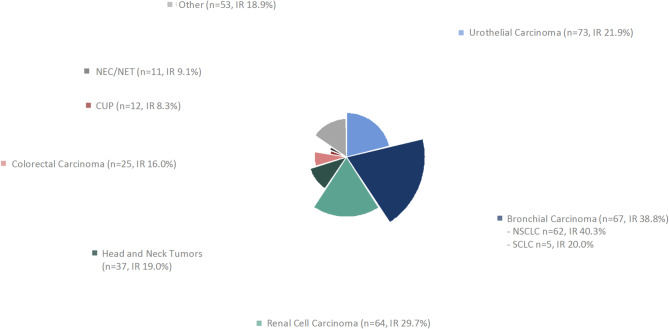
Distribution of tumor entities included in the study cohort. Each segment corresponds to a distinct tumor type. The darker inner segments represent the proportion of initial responders within each respective tumor entity. IR: initial responders; NEC: neuroendocrine carcinoma; NET: neuroendocrine tumor; CUP: cancer of unknown primary; HNSCC: head and neck squamous cell carcinoma.

We retrospectively identified 368 patients who received treatment with ICIs in the interdisciplinary CCCMunich^***LMU***^ outpatient clinic between January 2015 and November 2020. Of these, 26 patients were excluded because they continued ICI treatment elsewhere after the first application and no further clinical data was available. The median age at initial diagnosis of malignant disease of the analysed 342 patients was 63.5 years (range 19–88) and the median age at time of treatment initiation with ICI was 67.0 years (range 25–89). The population consisted of more male patients (67.8%) than female patients (32.2%). The most common entities were urothelial carcinoma (21.3%) and bronchial carcinoma, including NSCLC and SCLC (19.6%), followed by renal cell carcinoma (18.7%), and head and neck tumors (10.8%) (Fig. 1, Table S1). Most patients received ICIs for metastatic disease (stage IV 95.0%), only few patients were treated in earlier stages (stage II 0.3%; stage III 4.7%). Of all patients, 54 (15.8%) presented with a medical history of autoimmune disorders, most commonly thyroid disorders (71.4%) treated with hormonal replacement or anti-thyroid medication. Only three patients received low-dose systemic steroids at time of ICI start for rheumatoid arthritis, polymyalgia rheumatica, and immune thrombocytopenia, respectively. The ICIs used were mainly Pembrolizumab (50.3%), Nivolumab (31.9%) and Nivolumab combined with Ipilimumab (Nivo/Ipi) (9.6%). Only few patients received Atezolizumab (5.6%), Durvalumab (0.9%), Durvalumab combined with Tremelimumab (0.9%), Avelumab (0.6%) and Tremelimumab (0.3%). Most patients received only ICIs without other agents (75.1%), fewer patients received an ICI combined with chemotherapy or a tyrosine kinase inhibitor (24.0%). The median number of ICI infusions per patient was 10 (range 1–86) and the median treatment duration amounted to 6.9 months (range 0–58). Main reasons for treatment termination were progression of disease (40.4%), adverse events (10.8%), clinical deterioration (8.2%), and death (4.1%). At end of follow-up, 23.1% of patients were still receiving ICI treatment. Median follow-up time, estimated using the reverse Kaplan–Meier method, was 20.2 months.

### Outcome

**Fig. 2:**
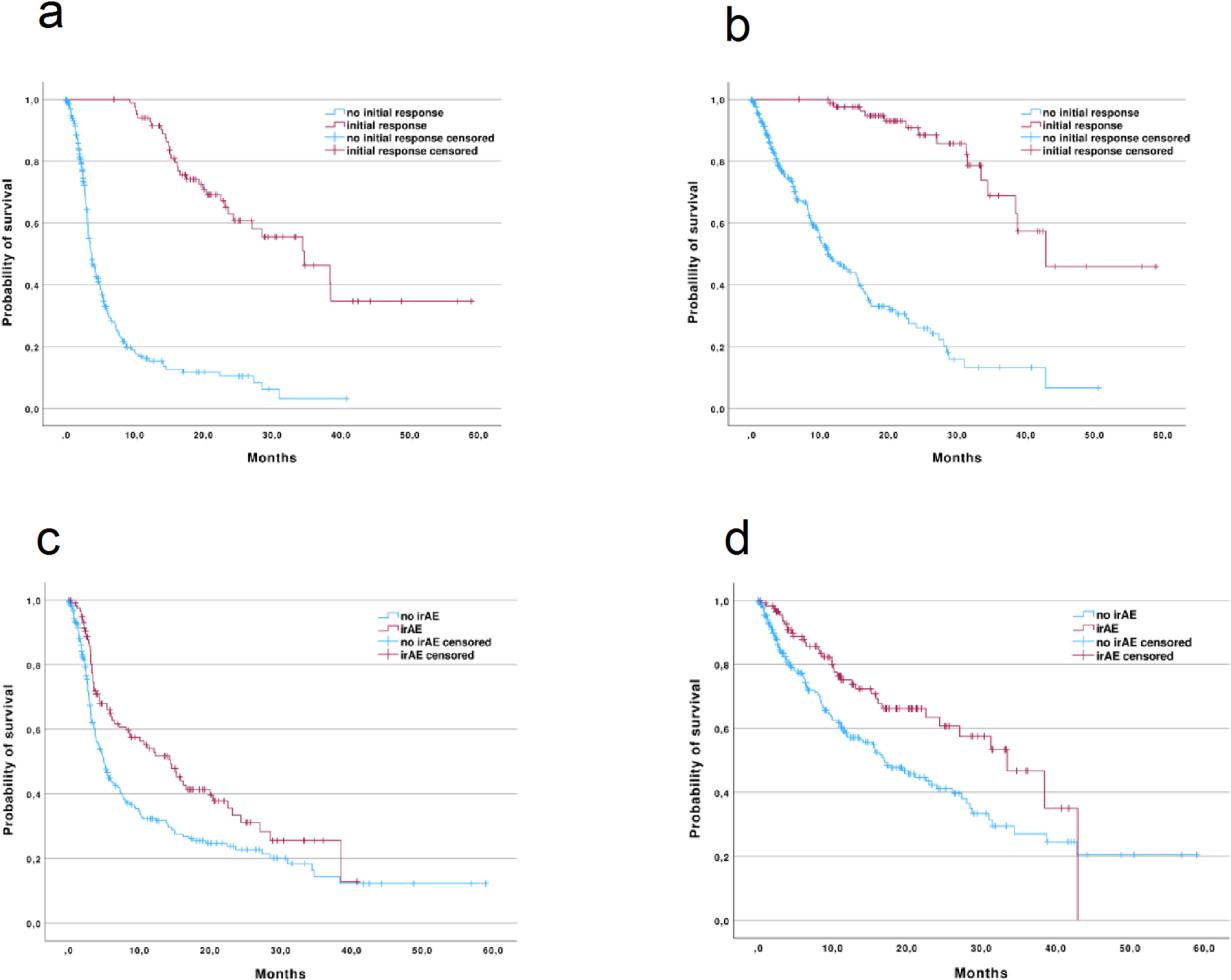
Kaplan–Meier curves for PFS (**a**) and OS (**b**), stratified by initial response status. Kaplan–Meier curves for PFS (**c**) and OS (**d**), stratified by occurrence of irAE. IRAE: immune related adverse event.

The overall initial responder rate was 24.6%, with male patients having slightly higher initial responder rates than female patients (26.7% vs 20.0%). In the more common entities with more than 20 cases in our cohort (urothelial carcinoma, bronchial carcinoma, renal cell carcinoma, head and neck tumors, colorectal carcinoma), the highest rates of initial response were observed in bronchial carcinoma (38.8%) and renal cell carcinoma (29.7%). An overview can be found in Fig. 1. As expected, median PFS was significantly longer in patients who responded initially than in patients without initial response (34.7 months vs. 3.8 months; p < 0.001) (Fig. 2a). This also translated into prolongation of median overall survival (OS) (43.0 months vs 11.1 months; p = 0.001) (Fig. 2b). Longest PFS in the more common entities was observed in patients with renal cell carcinoma (15.8 months; 95% CI 8.7–22.9) and NSCLC (12.1 months; 95% CI 7.1–17.1). Shortest median PFS was seen in patients with colorectal cancer (4.3 months; 95% CI 2.0–6.6).

There was a numerical difference between mPFS in male and female NSCLC patients, with males experiencing a longer mPFS (12.4 months vs 7.2 months). However, the difference was not statistically significant (p = 0.662), and also did not translate into longer OS (27.0 months vs 26.1 months; p = 0.859). There were no dependencies between age or sex and PFS in the other entities.

### Adverse events

**Fig. 3:**
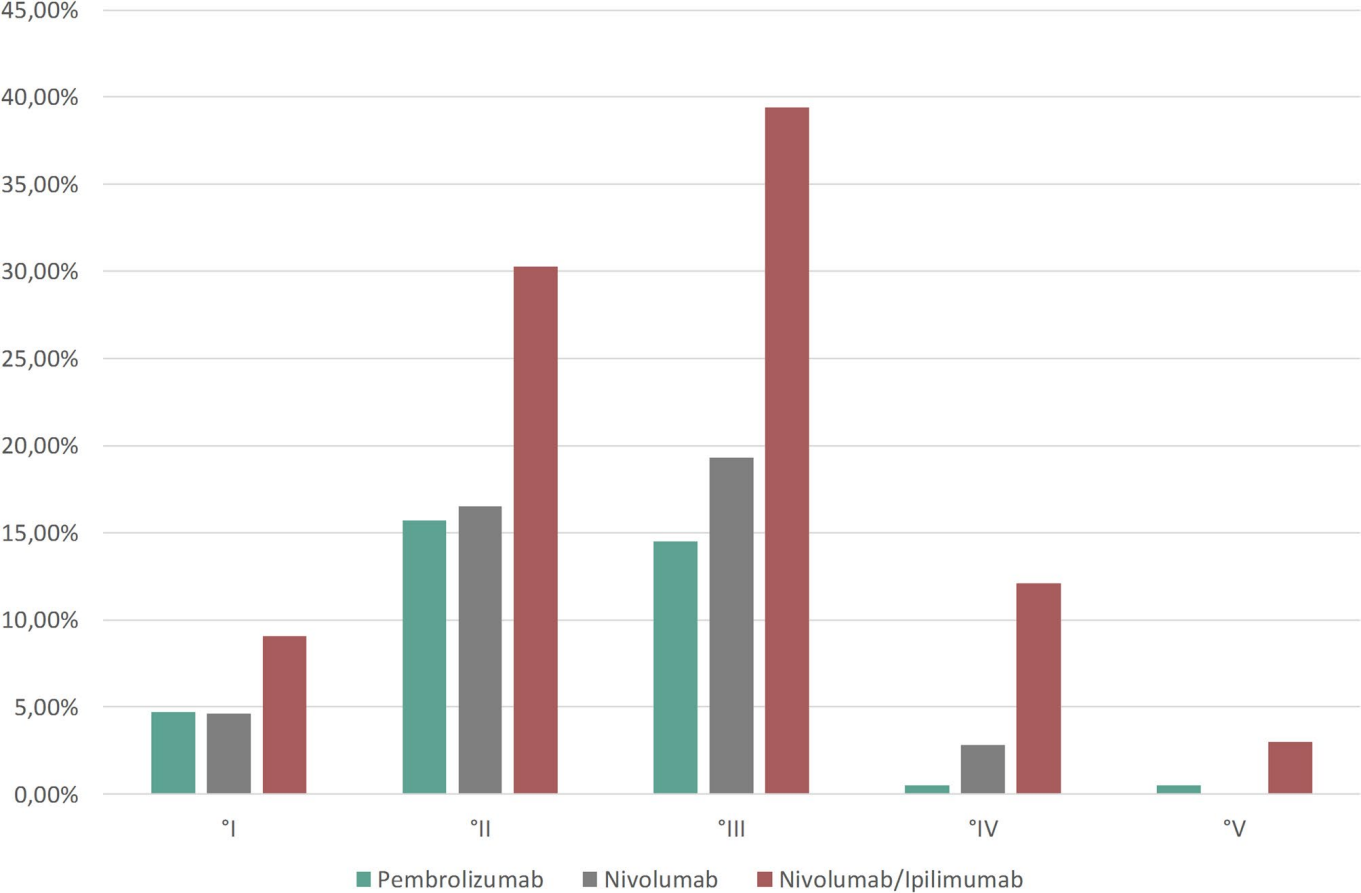
Frequency of adverse events observed under immunotherapies Pembrolizumab, Nivolumab, and Nivolumab/Ipilimumab, categorized by severity grade.

IrAEs were observed in 118 (34.5%) patients. Of these, 11.6% were defined as mild according to CTCAE (Common Terminology Criteria for Adverse Events) scaling, 39.0% as moderate, 41.8% as severe, 6.2% as life threatening, and 1.4% as fatal. Patients treated with double checkpoint inhibition with Nivo/Ipi experienced irAEs more frequently and at higher grades than patients treated with Pembrolizumab or Nivolumab monotherapy. However, as assessed by the Kruskal–Wallis test, the differences in grades between the groups were not statistically significant (p = 0.18) (Fig. 3). IrAEs occurred in male and female patients at similar rates (35.8% vs 31.8%, respectively). The rate of irAEs in patients with and without pre-existing autoimmune disorders was comparable (35.2% and 34.5%, respectively) as well. There was no statistically significant difference between the irAE grades of the two groups (Mann Whitney U test, p = 0.774). Furthermore, there was no statistically significant association between age and occurrence of irAEs (p = 0.14). In 34 patients (28.8%) with irAEs, treatment had to be discontinued, in 48 patients (40.7%), ICI treatment was paused temporarily and then restarted after irAE improvement. Of these 48 patients who restarted ICI after resolving or improving of the irAEs, only 11 patients ultimately had to terminate ICI treatment due to irAEs, the other patients continued ICI treatment until other reasons for termination occurred.

The main side effects were thyroiditis (8.8% of all patients), pneumonitis (7.0% of all patients), hepatitis (6.7% of all patients), colitis (5.8% of all patients), and dermal reactions (5.0% of all patients). Rare irAEs included encephalitis, vasculitis, stomatitis, and adrenal insufficiency (each occurred in 1 patient). The two fatal irAEs were colitis and pneumonitis, one occurred in a patient with history of an autoimmune disorder, and one in a patient without. An overview on the observed irAEs can be found in table S2.

Regarding irAEs and outcomes, patients who experienced irAEs had a significantly longer median PFS and OS than patients without irAEs (14.5 months vs 5.0 months; p = 0.002 and 33.5 months vs 17.0 months; p = 0.002, respectively) (Fig. 2c–d). There was no statistically significant difference in PFS depending on the grade of the irAEs (p = 0.787). Interestingly, in renal cell carcinoma, patients experiencing irAEs did not have a notably longer median PFS than patients without irAEs (15.8 vs. 15.0 months, p = 0.755).

### Analysis of laboratory results within the IR and INR groups

**Fig. 4:**
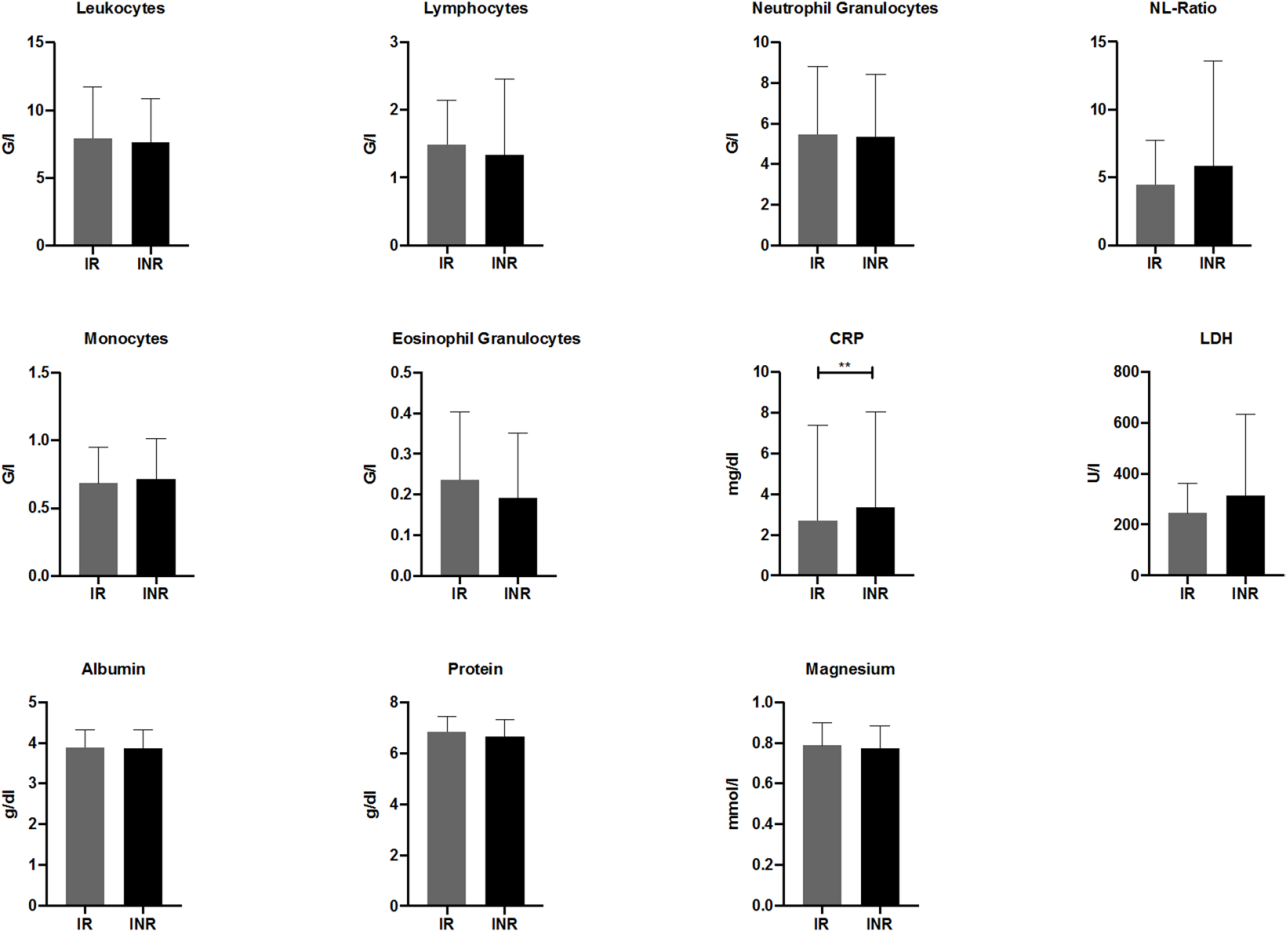
Baseline laboratory parameters in the initial responder and the initial non-responder group before initiation of ICI. IR: initial responders; INR: initial non-responders; NL-ratio: neutrophil–lymphocyte ratio; CRP: C-reactive protein; LDH: lactate dehydrogenase.

We analysed various laboratory parameters measured before the start of ICI therapy to assess whether any of them have predictive value for the patients’ initial response to treatment. A significant difference was identified for the parameter CRP (p = 0.047); higher levels prior to the start of ICI treatment were associated with INR (Fig. 4). Tendencies, although not statistically significant, were also observed for the parameters LDH (lactate dehydrogenase), NL-ratio, and eosinophils; elevated LDH (p = 0.3617) and NL-ratio (p = 0.3055), as well as a reduced count of eosinophils (0.1252), were observed in the INR group.

### Prognostic value of laboratory results

**Fig. 5:**
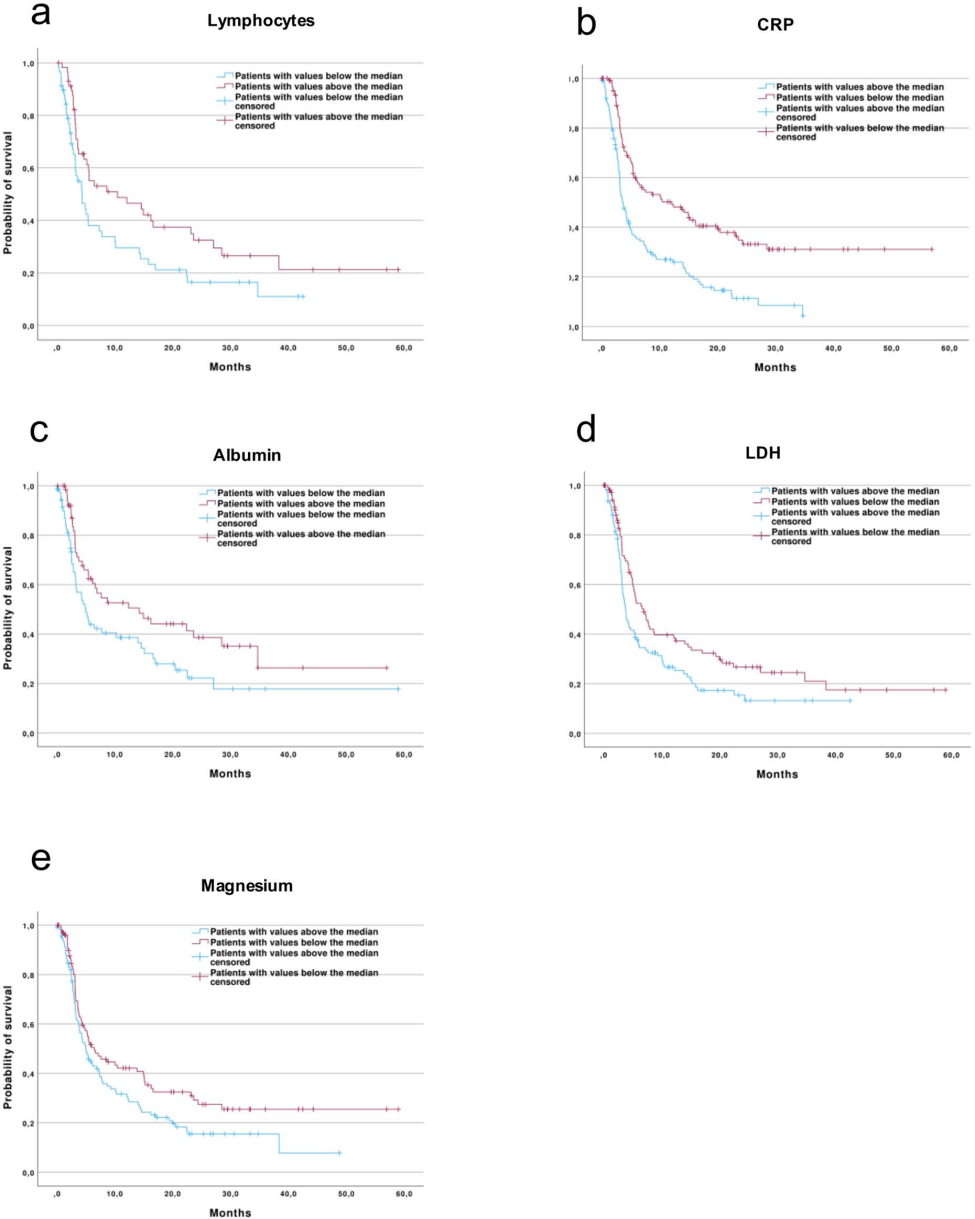
Kaplan–Meier curves for PFS stratified by baseline laboratory values above or below the median. CRP: C-reactive protein; LDH: lactate dehydrogenase.

In the next step, we examined the laboratory parameters prior to the start of ICI therapy in relation to PFS. We identified several parameters that were associated with PFS: For the parameters lymphocytes, albumin, and magnesium, values above the median were associated with significantly longer PFS. In contrast, for the parameters CRP and LDH, values above the median were associated with shorter PFS.

(Fig. 5a–e). To assess the potential impact of these parameters not only in the entire cohort but also in specific entities, the relationship between the parameters and PFS were analysed in entities with a sample size of more than ten patients. Particularly in the group of head and neck tumors, parameters were identified that showed an association with PFS: Leukocyte, neutrophil, and CRP-albumin ratio values above the median were associated with shorter PFS (Fig. S1a-c). In patients with renal cell carcinoma, a similar association with shorter PFS was observed for elevated monocyte counts (Fig. S1d).

### Predictive models for the risk of disease progression

The results of the Cox proportional hazards models obtained through forward selection based on the AIC and BIC criteria are presented in Table 1. Albumin and lactate dehydrogenase were selected in both models and were the only variables included in the BIC-based model. In both models, higher albumin and lactate dehydrogenase levels were associated with longer PFS.

**Table 1:** Hazard ratios from Cox proportional hazards models obtained by forward selection based on AIC and BIC.

	Hazard ratio (AIC model)	Hazard ratio (BIC model)
Albumin	0.647	0.576
Lymphocytes	0.726	
Lactate dehydrogenase (by 1000 U/L increase)	2.064	2.259
Monocytes	1.565	

The models demonstrated poor discriminatory ability, with concordance index (C-index) values of 0.574 (AIC model) and 0.563 (BIC model). Similarly, the Brier scores of 0.167 (AIC model) and 0.172 (BIC model) indicated poor to moderate calibration.

In the AIC-based model, additional predictors were selected: higher lymphocyte counts were associated with longer PFS, while higher monocyte counts were associated with shorter PFS.

## Discussion

This retrospective cohort study analysed patients treated with ICI in the interdisciplinary CCCMunich^*LMU*^ outpatient clinic over a five-year period. The patient cohort consists of patients treated in an early phase of ICI approvals by the European Medicines Agency. Therefore, the majority of the included entities are urothelial carcinoma, bronchial carcinoma, renal cell carcinoma, and head and neck tumors. 95% of the patients suffered from metastatic disease, and most patients were treated with Pembrolizumab or Nivolumab, which largely corresponds to the approval status in the years 2015–2020^18^.

Regarding the outcome, we decided to analyse the cohort based on the initial response, defining this as the response in the second radiological staging approximately six months after ICI initiation. This timepoint was chosen because the onset of action under immunotherapy is sometimes only observed after more than three months in radiologic imaging^19^. Regarding PFS, we observed a significant difference between the two groups, which was expected considering the definition of the INR group as having progressive disease in the second radiologic staging. Interestingly, this also translated into a significantly longer OS in initial responders, suggesting that once an initial response occurred, these patients benefited from immunotherapy over a prolonged period. This phenomenon of durable responses in a subgroup of treated patients has been described for ICI before, as opposed to targeted treatments which lead to regressions in most patients exhibiting the molecular target, however then lose efficacy due to development of treatment resistance^20,21^. In a cohort described by Noronha and colleagues, response rates in patients receiving checkpoint inhibitors for the treatment of non-melanoma solid tumors were higher than in our cohort, however median PFS and OS were significantly shorter^22^. Head and neck cancers in our cohort showed lower initial response rates, consistent with a study by Patil et al., where nivolumab achieved an overall response rate (ORR) of only 19.5% and a median PFS of 2.27 months in a heavily pretreated population^23^. Our data also showed no statistically significant sex-based differences in PFS or OS. This is consistent with a meta-analysis by Wallis and colleagues, which found no significant association between patient sex and survival outcomes under ICI treatment^24^. In summary, our findings support existing real-world evidence that ICIs offer meaningful and often durable benefits in a subset of patients with solid tumors.

An important issue in ICI therapy is the occurrence of immune-mediated side effects. In our cohort, irAEs occurred in approximately one-third of the patients. This is in line with previous data from other studies^25,26^. It is known that toxicity is increased when dual ICI therapy is administered^27^ which was observed in our study as well. Consistent with known data, our patient cohort experienced mainly gastrointestinal irAEs, thyroiditis, and cutaneous side effects^26,28^. A frequently discussed topic is the treatment rechallenge after occurrence of an irAE under ICI. In our cohort, this rechallenge was performed in 48 patients, with only 11 patients experiencing another irAE that led to treatment discontinuation, suggesting that a rechallenge may be evaluated by the treating physician and in accordance with the patient’s preference. Notably, patients who experienced an irAE had a significantly longer PFS and OS, than patients who did not experience any side effects from the treatment. This confirms findings by Mehra and colleagues, who described an association between irAE occurrence and improved survival in a cohort analysis of 229 patients treated with ICI^29^.

In the analysed cohort, patients with pre-existing autoimmune disorders did not experience irAEs more frequently than patients without pre-existing autoimmune disorders. Since most pre-existing autoimmune disorders were related to the thyroid, and only three patients were receiving low-dose systemic steroids when starting ICI treatment, no assumptions can be made regarding the occurrence of irAEs in patients with active, uncontrolled autoimmune disorders. However, in patients with a history of autoimmune disorders, which are currently inactive or in patients with thyroid disorders treated with hormonal replacement or anti-thyroid medication, our study did not provide any evidence that ICIs should not be applied, if indicated. These results add to the evidence of previous studies suggesting an acceptable safety profile of ICI in patients with pre-existing autoimmune disease^30,31^.

Predictive and prognostic markers in the peripheral blood have already been investigated in some studies. It has been shown that high levels of lymphocytes correlate with prolonged survival in patients with solid tumors treated with ICI^32^, which we were also able to demonstrate in our study. Similarly, we showed that higher serum albumin is associated with longer survival, which has also been confirmed in other studies^32^. Markers that were associated with shorter survival in our study included LDH and CRP. These markers have also been partially described across different entities as well as in specific entities, such as NSCLC and malignant melanoma^32–34^. A marker that has also gained attention is the neutrophil-to-lymphocyte ratio (NL ratio). A low NL ratio at the start of therapy has been found to correlate with better outcomes and prolonged survival, while a high NL ratio was associated with worse clinical outcome^35,36^. Our data showed a high NL ratio in the INR group, which aligns with previous findings. Additionally, we measured a reduced eosinophil count at the start of therapy in the INR group. This is consistent with published data that have shown that the level of eosinophils correlates with survival across different entities treated with ICI^29^. In summary, we were able to identify several markers in the peripheral blood that may correlate with survival under ICI. However, the integration of these parameters did not allow for the development of a valid predictive or prognostic score. Recently, a machine learning model was successfully developed that could reliably predict overall survival using only routine blood tests and clinical data. With this so-called SCORPIO model, it was possible to achieve a predictive power superior to that of PD-L1 expression or TMB^37^, indicating a possible use of machine learning models for response prediction in the future.

Some limitations to this study need to be noted: Because the timing of irAEs was not documented at the time of data collection, we were unable to account for potential guarantee-time bias when assessing the association between irAE occurrence and survival outcomes. Consequently, time-dependent Cox or landmark analyses could not be performed. Future prospective studies should ensure detailed recording of irAE onset times to enable robust causal inference regarding the prognostic impact of irAEs. Since this analysis reflects a single-center experience from our interdisciplinary outpatient clinic, some tumor entities such as malignant melanoma, that are commonly treated with ICIs, but not treated on our interdisciplinary ward, are not represented. Further, the retrospective nature of the study and the analysis of an inhomogeneous patient collective regarding diagnosis, line of therapy, and combination treatments, complicate the interpretation of the data. Additionally, patients who continued ICI treatment at other centers after the first dose were not captured in our dataset, which could bias our findings by underestimating toxicity or overestimating efficacy. Furthermore, the analysed laboratory parameters were not available from all patients, therefore a smaller data set had to be used for the evaluation of predictive laboratory markers. This could influence the reliability of the model, and may be a reason for predictive markers already described in the literature only showing a tendency without statistical significance in our analysis. The incompleteness of the data also complicated the integration of the parameters into a predictive or prognostic score. Nevertheless, it was possible to identify certain parameters which should be investigated further in a prospective study.

## Supplementary Information

Below is the link to the electronic supplementary material.

Supplementary Material 1.

## Data Availability

The datasets generated and analysed during the current study are available from the corresponding author upon reasonable request. The R code produced for the development and validation of the predictive models for progression-free survival, is available on GitHub under the following link: https://github.com/TimLandfarth/progression-free-survival.git.
