# Supplementary material for: Real-world analysis of immune checkpoint inhibitor efficacy and response predictors in patients treated at the CCCMunichLMU outpatient clinic
Source: Sci Rep. 2025 Dec 8;15:43269. doi: 10.1038/s41598-025-30220-0 (PMC12686437; doi:10.1038/s41598-025-30220-0)

Real-world analysis of immune checkpoint inhibitor efficacy and response predictors in patients treated at the CCCMunich^{LMU} outpatient clinic

Supplement

Table S1. Baseline characteristics of all included patients

**Tumor entities**	
Urothelial carcinoma	73 (21.3%)
Bronchial carcinoma	67 (19.6%)
NSCLC	62 (18.1%)
SCLC	5 (1.5%)
Renal cell carcinoma	64 (18.7%)
Head and neck cancer	37 (10.8%)
Colorectal carcinoma	25 (7.3%)
CUP	12 (3.5%)
NEC/NET	11 (3.2%)
Oesophageal carcinoma	9 (2.6 %)
Cholangiocellular carcinoma	7 (2.0%)
Hodgkin lymphoma	6 (1.8%)
Thymic carcinoma	4 (1.2%)
Pancreatic cancer	4 (1.2%)
Anal carcinoma	4 (1.2%)
Other lymphoma	3 (0.9%)
Melanoma	3 (0.9%)
Gastric cancer	3 (0.9%)
Sarcoma	2 (0.6%)
SCC	1 (0.3 %)
Prostate cancer	1 (0.3%)
Pseudomyxoma peritonei	1 (0.3%)
Urachal carcinoma	1 (0.3%)
Malignant germ cell tumor	1 (0.3%)
Peritoneal mesothelioma	1 (0.3%)
Glioblastoma	1 (0.3%)
Leukemia	1 (0.3%)
**Tumor stage**	

I	0 (0.0%)
II	1 (0.3%)
III	16 (4.7%)
IV	325 (95.0%)
**Age median (min-max)**	67.0 (25-89)
**Sex**	
female	110 (32.2%)
male	232 (67.8%)
**Patients with pre-existing autoimmune conditions**	54 (15.8%)

Table S2. Adverse events in patients experiencing irAEs (n=118)

Thyroiditis	30 (20.5%)
Pneumonitis	24 (16.4%)
Hepatitis	23 (15.8%)
Dermal reaction	17 (11.6%)
Colitis	20 (13.7%)
Arthritis	8 (5.5%)
Neurotoxicity	4 (2,7%)
Hypophysitis	4 (2,7%)
Myocarditis	2 (1,4%)
Diabetes mellitus type I	2 (1,4%)
Renal dysfunction	2 (1,4%)
Myositis	2 (1,4%)
Allergic reaction	2 (1,4%)
Toxic cardiomyopathy	2 (1,4%)
Stomatitis	1 (0,7%)
Adrenal insufficiency	1 (0,7%)
Vasculitis	1 (0,7%)
Encephalitis	1 (0,7%)

Figure S1. Kaplan-Meier curves for PFS stratified by baseline laboratory values of leukocytes (a), neutrophils (b), and CRP/Albumin ratio (c) above or below the median in head and neck cancer. Kaplan-Meier curve for PFS stratified by baseline monocyte count above or below the median in renal cell carcinoma (d). CRP: C-reactive protein.

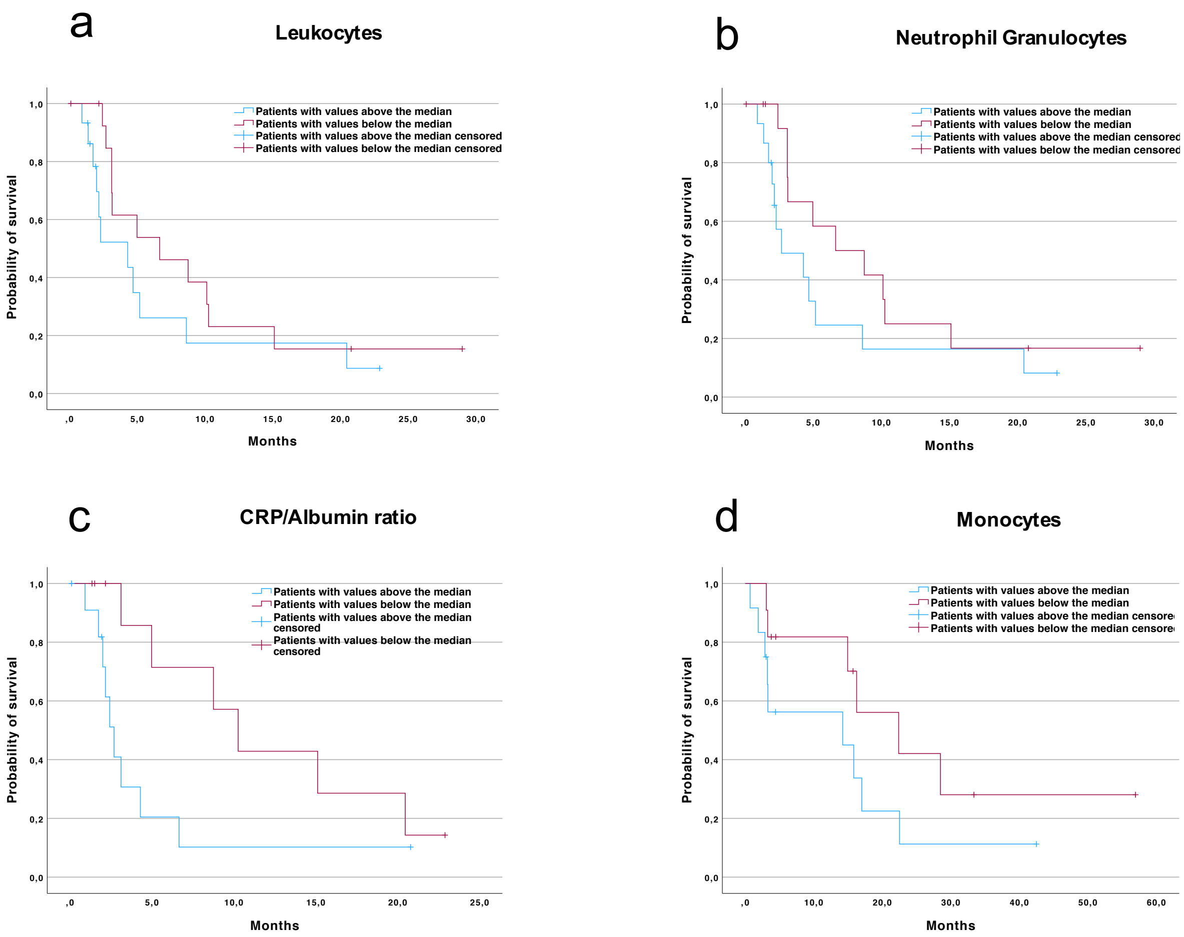

Supplement: Supplementary file 1 — Supplementary Material 1. [file 41598_2025_30220_MOESM1_ESM.pdf]
